# Dopamine D2 and GABA(A) Receptors Differentially Regulate Ethanol-Induced Aversion and Reward Through Corticolimbic Circuits

**DOI:** 10.3390/ijms27114987

**Published:** 2026-05-30

**Authors:** Cheng En Wu, Yu Cheng Lin, Zhi-Yue Gao, Anna Kozłowska, Cai-N Cheng, Chi-Wen Wu, Andrew Chih Wei Huang

**Affiliations:** 1 Department of Psychology, Fo Guang University, Yilan County 26247, Taiwan; a741123369987@gmail.com (C.E.W.); a0918764764@gmail.com (Y.C.L.); shps90060801@yahoo.com.tw (C.-N.C.); 2 Yuanshan Branch, Taipei Veterans General Hospital, Yilan County 264018, Taiwan; com00000com@yahoo.com.tw; 3 Department of Human Physiology and Pathophysiology, Collegium Medicum, University of Warmia and Mazury, Warszawska Av, 30, 10-082 Olsztyn, Poland; kozlowska.anna@uwm.edu.pl; 4 Department of Pharmacy, Keelung Hospital, Ministry of Health and Welfare, Keelung City 201, Taiwan; pedophillia0722@gmail.com

**Keywords:** dopamine D2 receptor, GABA(A) receptor, corticolimbic circuits, ethanol intoxication, aversion, reward

## Abstract

Ethanol produces both aversive and rewarding effects during the intoxication phase; however, the receptor-specific pharmacological mechanisms and neural circuits underlying this paradox remain poorly defined. The present study investigated how dopamine D2 and GABA(A) receptor systems differentially regulate ethanol-induced aversion and reward at behavioral and neural circuit levels. Rats received systemic administration of the dopamine D2 receptor agonist apomorphine, the GABA(A) receptor agonist muscimol, or the GABA(A) receptor antagonist bicuculline prior to ethanol conditioning. Ethanol-induced aversion and reward were assessed using conditioned taste aversion (CTA) and conditioned place preference (CPP), respectively, and neural activation was examined using c-Fos immunohistochemistry in the medial prefrontal cortex, amygdala, and hippocampus. Apomorphine potentiated ethanol-induced CTA while suppressing ethanol-induced CPP. In contrast, bicuculline attenuated ethanol-induced CTA and abolished ethanol-induced CPP, whereas muscimol enhanced aversive CTA and converted ethanol-induced CPP into conditioned place aversion. During CTA, apomorphine predominantly changed c-Fos expression in amygdalar and hippocampal subregions, whereas GABA(A) receptor manipulation altered activity within the medial prefrontal–amygdala–hippocampal network. During CPP, dopamine D2 receptor activation enhanced neural activity in the medial prefrontal cortex and hippocampus while suppressing central amygdala activity, whereas GABA(A) receptor modulation reduced prefrontal activation and enhanced amygdalar and hippocampal engagement. Altogether, these findings reveal receptor-specific and context-dependent corticolimbic mechanisms through which dopamine D2 and GABA(A) receptors differentially regulate ethanol-induced aversive and rewarding states during acute intoxication.

## 1. Introduction

Substances of abuse, including ethanol [1,2], morphine [3,4], and amphetamine [5,6], exert complex neurobiological effects, eliciting both aversive (e.g., vomiting, avoidance) and rewarding (e.g., craving) features, which contribute to the approach and/or avoidance behaviors in drug addiction. Acute ethanol consumption often induces rewarding and aversive effects during the intoxication phase; however, chronic ethanol exposure frequently leads to negative reinforcement, particularly during the withdrawal phase following cessation [7,8]. However, there exists a body of research investigating the immediate rewarding and aversive effects of ethanol following acute and short-term administration. These studies aim to model the early stages of alcohol use disorder (AUD) during the intoxication phase, rather than focusing on withdrawal symptoms [2,5,9,10]. At the neuropharmacological level, ethanol engages dopaminergic and GABAergic receptor systems that converge on corticolimbic circuits to regulate aversive and rewarding behavioral states during intoxication.

To our knowledge, the dopamine and GABA systems differentially modulate ethanol-induced aversion and reward. For example, emerging evidence regarding the potential underlying neural mechanisms suggests a critical role for the interplay between dopaminergic and GABAergic systems in modulating voluntary ethanol consumption and binge drinking [11]. Indeed, both of these systems have been implicated in mediating ethanol-induced aversion [12] and reward [13,14]. For instance, a review paper reported that a GABA(A) receptor antagonist increased both ethanol-induced CPP and CTA in mice; however, it only attenuated CTA (but not CPP) in rats [14]. This highlights species-specific differences or complex modulatory roles. While intraperitoneal injections of D2 antagonist haloperidol and D1 antagonist SCH-23390 were both found to block ethanol-induced CTA [12], the roles of these receptors in ethanol-induced reward (CPP) appear to be distinct, with later research finding that SCH-23390 attenuated CPP acquisition, whereas an intraperitoneal injection of D2 antagonist raclopride had no effect [13]. In summary, how the dopamine and GABA systems differentially modulate ethanol aversion and reward remains uncertain. Therefore, the precise roles of D2 receptors in mediating ethanol’s aversive (CTA) and rewarding (CPP) effects require further elucidation. A re-examination of GABA(A) receptor modulation of both ethanol-induced aversion and reward is also warranted, given the inconsistent findings across previous studies.

Some specific brain areas have been implicated in excessive ethanol consumption. For example, converging evidence suggests that the impairment of cognitive functions caused by acute or chronic intermittent ethanol administration is mediated by the medial prefrontal cortex (mPFC) [15]. This impairment can disrupt the mPFC’s inhibitory control over negative emotional processing within the amygdala. This results in hyperactivity in the central amygdala (CeA), which is implicated in driving the excessive alcohol consumption that contributes to alcohol dependence [15]. fMRI studies have demonstrated significant associations between cognitive dysfunctions, prefrontal cortex impairments, and an inability to effectively regulate cravings and negative emotional responses in individuals with AUD [16]. The hippocampus plays a crucial role in spatial learning and memory [17], and both acute and chronic ethanol intoxication have been shown to impair memory functions [18] and synaptic plasticity [19], which are both critically regulated by hippocampal neural activity. It is therefore plausible that acute ethanol intoxication impairs learning and memory via alterations in the hippocampus and its interconnected structures [20]. Given the established roles of these neural substrates in addiction-related behaviors [21,22], the present study utilized c-Fos immunohistochemistry to investigate the involvement of specific subregions within the mPFC [e.g., cingulate cortex 1 (Cg1), prelimbic cortex (PrL), infralimbic cortex (IL)], amygdala [e.g., basolateral amygdala (BLA), central amygdala (CeA)], and hippocampus [e.g., CA1, CA2, CA3, and dentate gyrus (DG)] in mediating ethanol-induced aversion (CTA) and reward (CPP).

Building upon the above, the present study aimed to address the following key questions: Can acute ethanol administration simultaneously induce both aversive (CTA) and rewarding (CPP) effects? How does the dopamine D2 agonist, apomorphine (APO), modulate ethanol-induced CTA and CPP? Do the GABA(A) receptor antagonist, bicuculline, and the GABA(A) receptor agonist, muscimol, differentially alter ethanol-induced aversion (CTA) and reward (CPP)? Are specific subareas of the mPFC (Cg1, PrL, IL), amygdala (BLA, CeA), and hippocampus (CA1, CA2, CA3, DG) involved in ethanol-induced aversion and reward? Do these brain areas contribute to the modulation of ethanol-induced aversion and reward by D2 and GABA(A) receptors during intoxication?

## 2. Results

### 2.1. Experiment 1: Dopamine and Alcohol-Induced Aversive CTA and Rewarding CPP Behaviors

**Figure 1:**
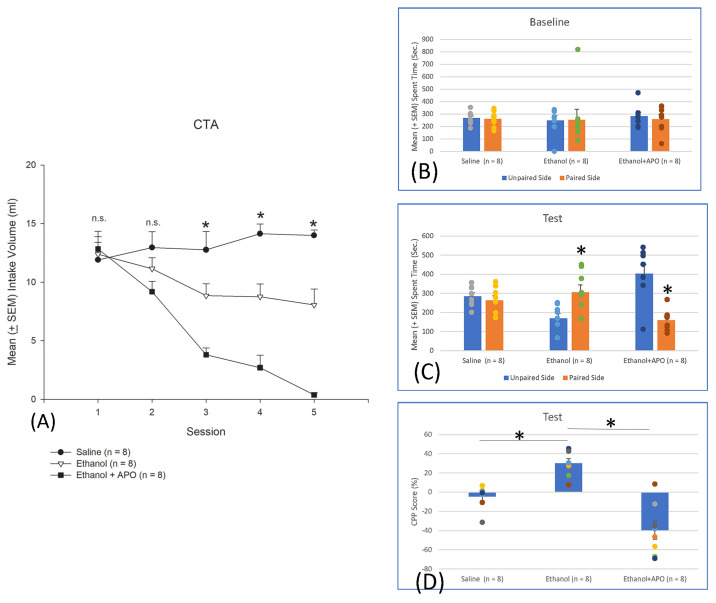
Dopamine system’s contribution to ethanol-induced aversion (CTA) and reward (CPP). (**A**) Mean (±SEM) intake volume (mL) of 0.1% saccharin solution for the Saline, Ethanol, and Ethanol + APO groups (n = 8 per group) across sessions 1–5. (**B**,**C**) Mean (±SEM) time spent (seconds) in the unpaired and paired sides for the Saline, Ethanol, and Ethanol + APO groups (n = 8 per group) during the Baseline (**B**) and Test (**C**) phases. (**D**) Conditioned place preference (CPP) score (%) for the Saline, Ethanol, and Ethanol + APO groups (n = 8 per group) in the test phase. (*): significant differences (*p* < 0.05). (n.s.): non-significant differences.

These results indicate that ethanol administration induced CTA (for group factors of group [F(2, 21) = 21.76, *p* < 0.05] and session [F(4, 84) = 15.42, *p* < 0.05], as well as a significant group × session interaction [F(8, 84) = 10.71, *p* < 0.05] using 3 × 5 mixed two-way ANOVA). High doses of APO, a dopamine agonist, enhanced ethanol-induced CTA, as the injections were associated with a decreased intake volume of saccharin solution (in sessions 3 (*p* < 0.05), session 4 (*p* < 0.05), and session 5 (*p* < 0.05) when comparing Saline and Ethanol groups following post hoc Tukey test), confirming the involvement of the brain’s dopamine system in ethanol-induced CTA (Figure 1A).

A dependent *t*-test was performed to investigate ethanol-induced CPP learning. During the baseline phase, ethanol did not induce CPP learning for the Saline (t(7) = 0.29, *p* > 0.05), Ethanol (t(7) = −0.05, *p* > 0.05), Ethanol + APO (t(7) = 0.53, *p* > 0.05) groups (using *t*-tests, no significant differences were observed between unpaired and paired sides for all groups (*p* > 0.05; Figure 1B)). Likewise, during the testing phase, the Saline group did not show CPP learning to compare the unpaired and paired sides (*p* > 0.05); The Ethanol group induced CPP learning due to time spent on the paired side compared to that of the unpaired side (t(7) = −5.01, *p* < 0.05). Time spent by the Ethanol + APO group on the paired side was significantly decreased compared to the unpaired side (t(7) = 4.35, *p* < 0.05) (Figure 1C).

Using CPP score (%) analysis by one-way ANOVA [F(2, 21) = 28.07, *p* < 0.05], the Ethanol group showed that the CPP score was higher than that of the Saline group (*p* < 0.05); however, the CPP score (%) of the Ethanol + APO group was significantly lower than that of the Ethanol group (*p* < 0.05) following post hoc Tukey tests (Figure 1D).

### 2.2. Experiment 1: Dopamine and Alcohol-Induced Aversive CTA and Rewarding CPP—Immunohistochemical Staining

#### 2.2.1. CTA Learning

**Figure 2:**
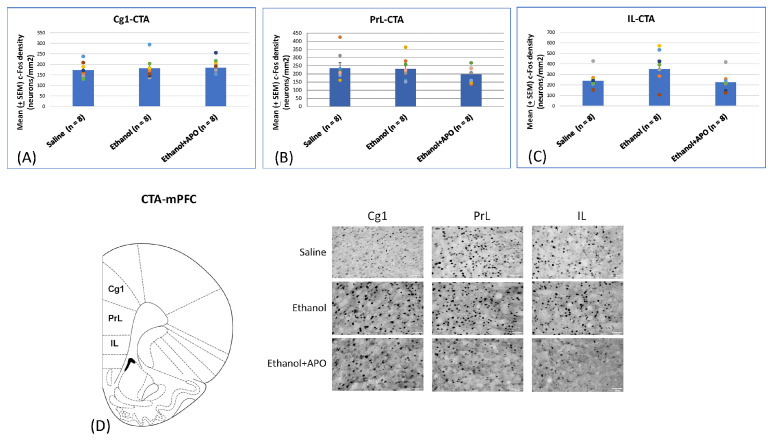
c-Fos density (neurons/mm^2^) in (**A**) Cg1, (**B**) PrL, and (**C**) IL of the mPFC in the dopamine system experiment for the Saline, Ethanol, and Ethanol + APO groups (n = 8 per group) following ethanol-induced aversion in CTA. (**D**) Representative cartoon brain map illustrating the locations of Cg1, PrL, and IL (**left panel**) and corresponding immunohistochemical staining images showing c-Fos expression (**right panel**).

**Figure 3:**
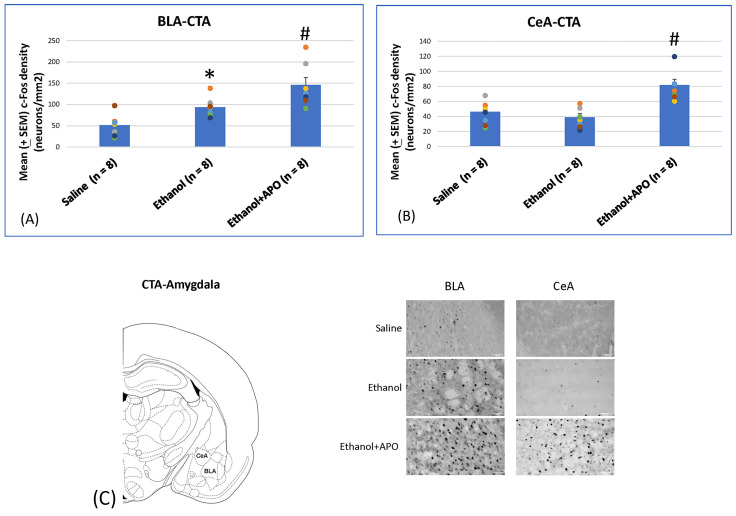
c-Fos density (neurons/mm^2^) in (**A**) BLA and (**B**) CeA in the dopamine system experiment for the Saline, Ethanol, and Ethanol + APO groups (n = 8 per group) after ethanol-induced aversion in CTA. (**C**) Representative cartoon brain map illustrating the locations of BLA and CeA (**left panel**) and corresponding immunohistochemical staining images showing c-Fos expression (**right panel**). (*) indicates significant differences compared to the Saline group. (#) indicates significant differences compared to the Ethanol group.

**Figure 4:**
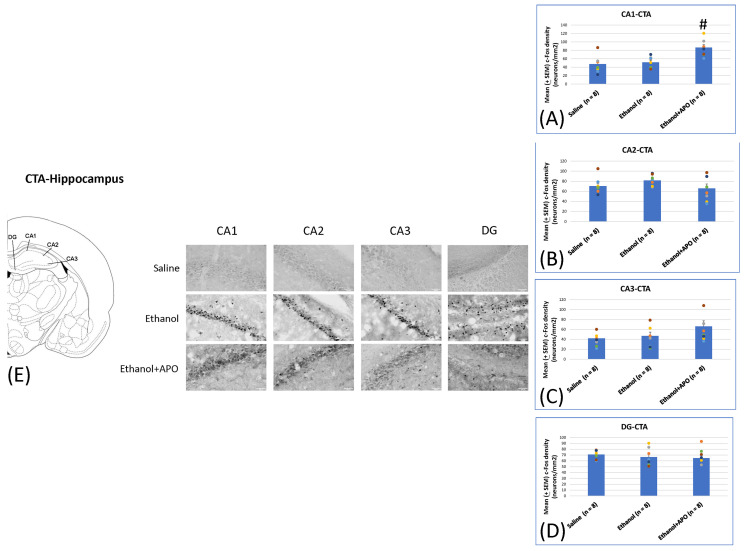
c-Fos density (neurons/mm^2^) in (**A**) CA1, (**B**) CA2, (**C**) CA3, and (**D**) DG of the hippocampus in the dopamine system experiment for the Saline, Ethanol, and Ethanol + APO groups (n = 8 per group) following ethanol-induced aversion in CTA. (**E**) Representative cartoon brain map illustrating the locations of CA1, CA2, CA3, and DG (**left panel**) and corresponding immunohistochemical staining images showing c-Fos expression (**right panel**). (#) indicates significant differences compared to the Ethanol group.

The results of c-Fos expression indicated no significant differences across groups in the Cg1 [F(2, 21) = 0.12, *p* > 0.05], PrL [F(2, 21) = 0.68, *p* > 0.05], or IL [F(2, 21) = 2.58, *p* > 0.05] following the CTA procedure (Figure 2A–C).

c-Fos expression in the Ethanol group was significantly increased compared to the Saline group (*p* < 0.05); moreover, c-Fos expression in the Ethanol + APO group was significantly increased compared to the Ethanol group (*p* < 0.05) following post hoc Tukey tests (Figure 3A). On the other hand, significant increases in c-Fos expression were also observed in the CeA for all groups [F(2, 21) = 13.45, *p* < 0.05]. This increase in c-Fos expression was significantly greater in the Ethanol + APO group compared to the Ethanol group (*p* < 0.05) following post hoc Tukey tests (Figure 3B).

c-Fos expression was significantly increased in the Ethanol + APO group when it was compared to the Ethanol group (*p* < 0.05) following post hoc Tukey tests (Figure 4A). However, no significant differences were observed in the CA2 [F(2, 21) = 1.70, *p* > 0.05], CA3 [F(2, 21) = 2.28, *p* > 0.05], or DG [F(2, 21) = 0.36, *p* > 0.05] using one-way ANOVA analysis for all groups (Figure 4B–D).

#### 2.2.2. CPP Learning

**Figure 5:**
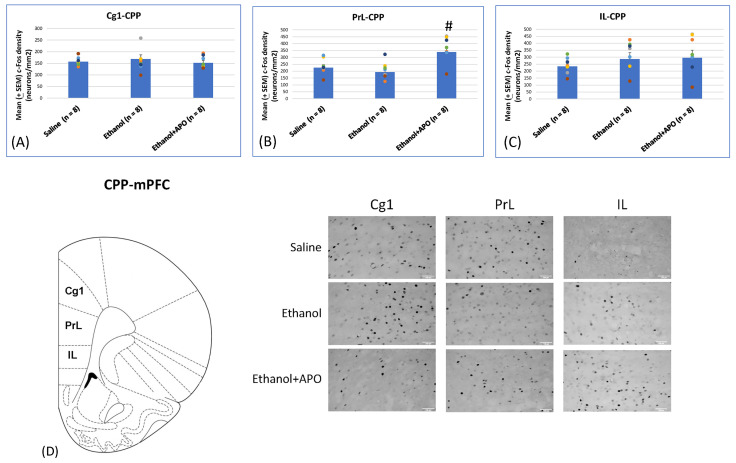
c-Fos density (neurons/mm^2^) in (**A**) Cg1, (**B**) PrL, and (**C**) IL of the mPFC in the dopamine system experiment for the Saline, Ethanol, and Ethanol + APO groups (n = 8 per group) after ethanol-induced reward in CPP. (**D**) Representative cartoon brain map illustrating the locations of Cg1, PrL, and IL (**left panel**) and corresponding immunohistochemical staining images showing c-Fos expression (**right panel**). (#) indicates significant differences compared to the Ethanol group.

After the ethanol-induced CPP learning, the PrL showed a significant increase in c-Fos expression in the Ethanol + APO group compared to the Ethanol group (*p* < 0.05; Figure 5B) following post hoc Tukey tests. However, no significant differences in c-Fos expression were observed in the Cg1 [F(2, 21) = 0.45, *p* > 0.05] or IL [F(2, 21) = 0.58, *p* > 0.05] (Figure 5A,C) after one-way ANOVA analysis.

**Figure 6:**
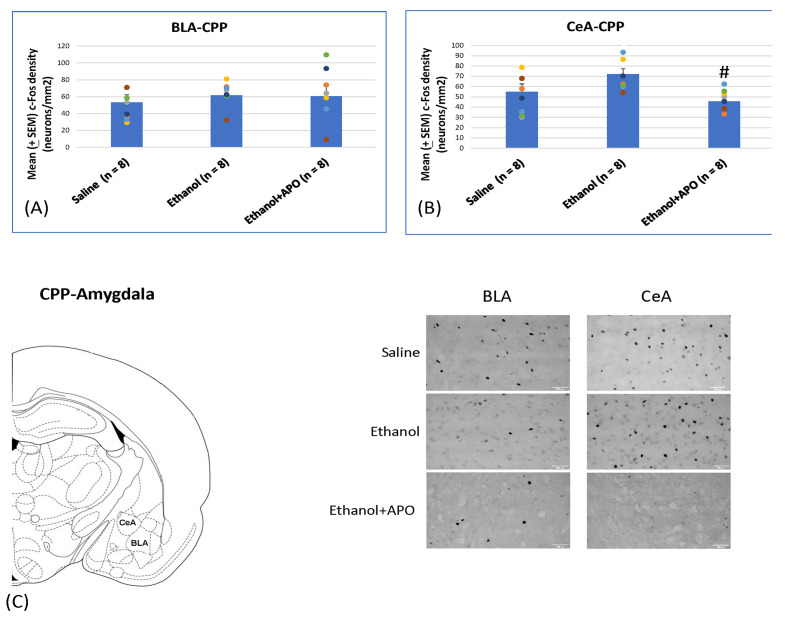
c-Fos density (neurons/mm^2^) in (**A**) BLA and (**B**) CeA in the dopamine system experiment for the Saline, Ethanol, and Ethanol + APO groups (n = 8 per group) following ethanol-induced reward in CPP. (**C**) Representative cartoon brain map illustrating the locations of BLA and CeA (**left panel**) and corresponding immunohistochemical staining images showing c-Fos expression (**right panel**). (#) indicates significant differences compared to the Ethanol group.

Significant differences in c-Fos expression were observed in the CeA following CPP learning [F(2, 21) = 4.96, *p* < 0.05]. Post hoc Tukey tests revealed that c-Fos expression was significantly lower in the Ethanol + APO groups compared to the Ethanol group (*p* < 0.05; Figure 6B). c-Fos expression in the Ethanol group was not significantly different from the Saline group (*p* > 0.05; Figure 6B). No significant differences in c-Fos expression were observed in the BLA [F(2, 21) = 0.26, *p* > 0.05; Figure 6A].

**Figure 7:**
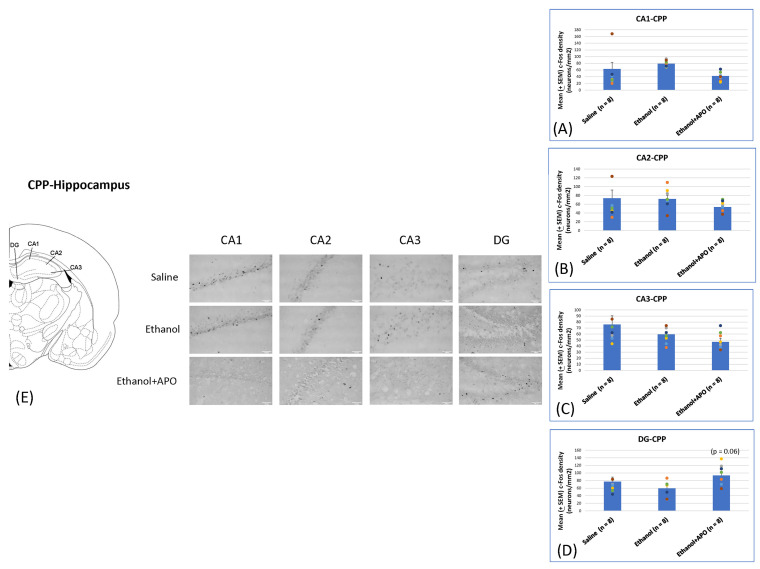
c-Fos density (neurons/mm^2^) in (**A**) CA1, (**B**) CA2, (**C**) CA3, and (**D**) DG of the hippocampus in the dopamine system experiment for the Saline, Ethanol, and Ethanol + APO groups (n = 8 per group) following ethanol-induced reward in CPP. (**E**) Representative cartoon brain map illustrating the locations of CA1, CA2, CA3, and DG (**left panel**) and corresponding immunohistochemical staining images showing c-Fos expression (**right panel**).

The results indicated no significant differences were observed in DG across groups [F(2, 21) = 2.92, *p* > 0.05], although the Ethanol + APO group showed a numerically higher c-Fos expression than the Ethanol group (*p* = 0.06) following the CPP learning procedure (Figure 7D). No significant differences were observed in the CA1 [F(2, 21) = 2.39, *p* > 0.05], CA2 [F(2, 21) = 0.80, *p* > 0.05], or CA3 [F(2, 21) = 2.41, *p* > 0.05] across groups (Figure 7A–C).

#### 2.2.3. Experiment 2: GABA and Alcohol-Induced Aversive CTA and CPP Behaviors

**Figure 8:**
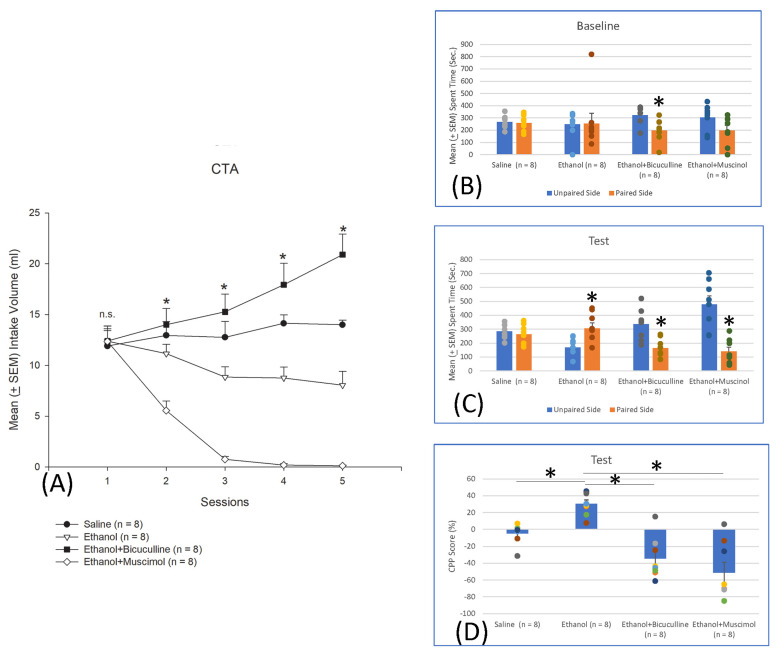
GABA system’s contribution to ethanol-induced aversion (CTA) and reward (CPP). (**A**) Mean (±SEM) intake volume (mL) of 0.1% saccharin solution for the Saline, Ethanol, Ethanol + Bicuculline, and Ethanol + Muscimol groups (n = 8 per group) across sessions 1–5. (**B**,**C**) Mean (±SEM) time spent (seconds) in the unpaired and paired sides for the Saline, Ethanol, Ethanol + Bicuculline, and Ethanol + Muscimol groups (n = 8 per group) during the baseline (**B**) and test (**C**) phases. (**D**) Conditioned place preference (CPP) score (%) for the Saline, Ethanol, Ethanol + Bicuculline, and Ethanol + Muscimol groups (n = 8 per group) during the test phase. (*) indicates significant differences (*p* < 0.05). (n.s.) indicates non-significant differences.

The results showed that the Ethanol group exhibited significantly decreased saccharin solution intake volumes compared to the Saline group in session 4 (*p* < 0.05) and session 5 (*p* < 0.05) following post hoc Tukey tests. Additionally, the Ethanol + Bicuculline group exhibited a significantly increased intake volume of saccharin solution compared to the Ethanol group during sessions 3 (*p* < 0.05), session 4 (*p* < 0.05), and session 5 (*p* < 0.05). The intake volume of the Ethanol + Muscimol group was significantly decreased compared to the Ethanol group during sessions 2 (*p* < 0.05), session 3 (*p* < 0.05), session 4 (*p* < 0.05), and session 5 (*p* < 0.05). These results confirm that ethanol administration induced the aversive effect during the CTA task. Injections of GABA(A) receptor antagonist, bicuculline, enhanced the saccharin solution intake volume and attenuated ethanol-induced CTA. Injections of GABA(A) agonist, muscimol, decreased the saccharin solution intake volume and enhanced ethanol-induced CTA. These results confirm the involvement of the brain’s GABA system in ethanol-induced CTA (Figure 8A).

During the baseline phase, no significant differences were observed between time spent in unpaired and paired sides for the Saline (t(7) = 0.29, *p* > 0.05), Ethanol (t(7) = −0.05, *p* > 0.05), and Ethanol + Muscimol (t(7) = 1.97, *p* > 0.05) groups; however, the Ethanol + Bicuculline group showed significant decreases in time spent in the paired sides compared to the unpaired sides during the baseline phase (t(7) = 3.02, *p* < 0.05; Figure 8B). During the test phase, the results showed that there were no significant differences between unpaired and paired sides for the Saline group during this phase (t(7) = 0.97, *p* > 0.05). Additionally, significant increases were observed between unpaired and paired sides for the Ethanol group (t(7) = −5.01, *p* < 0.05). Time spent in the paired side was significantly decreased compared to the unpaired side for the Ethanol + Bicuculline (t(7) = 3.83, *p* < 0.05) and Ethanol + Muscimol groups (t(7) = 3.87, *p* < 0.05; Figure 8C).

On the other hand, the CPP score (%) was significantly increased in the Ethanol group compared to the Saline group (*p* < 0.05) during the test phase. The CPP score (%) was significantly decreased in the Ethanol + Bicuculline (*p* < 0.05) and Ethanol + Muscimol (*p* < 0.05) groups compared to the Ethanol group following post hoc Tukey tests (Figure 8D).

### 2.3. Experiment 2: GABA and Alcohol-Induced Aversive CTA and Rewarding CPP—Immunohistochemical Staining

#### 2.3.1. CTA Learning

**Figure 9:**
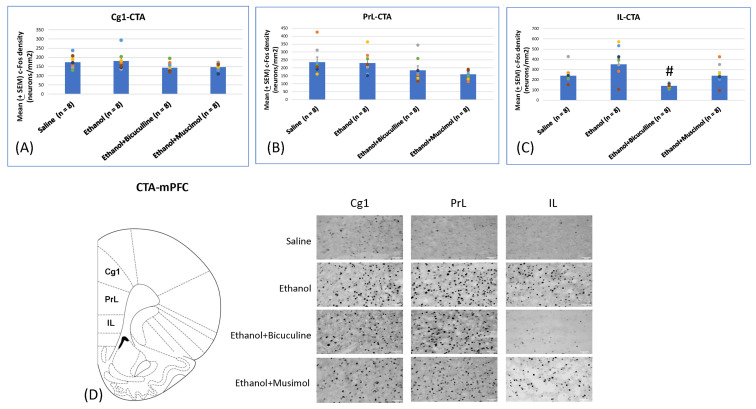
c-Fos density (neurons/mm^2^) in (**A**) Cg1, (**B**) PrL, and (**C**) IL of the mPFC in the GABA system experiment for the Saline, Ethanol, Ethanol + Bicuculline, and Ethanol + Muscimol groups (n = 8 per group) following ethanol-induced aversion in CTA. (**D**) Representative cartoon brain map illustrating the locations of Cg1, PrL, and IL (**left panel**) and corresponding immunohistochemical staining images showing c-Fos expression (**right panel**). (#) indicates significant differences compared to the Ethanol group.

To test c-Fos expression in the IL following the ethanol-induced CTA procedure, no significant differences were observed in the Saline group (*p* > 0.05) and the Ethanol + Muscimol (*p* > 0.05) compared to the Ethanol group. The Ethanol + Bicuculline group exhibited significantly decreased c-Fos expression compared to the Ethanol group (*p* < 0.05; Figure 9C) following post hoc Tukey tests. No significant differences in c-Fos expression were observed in the Cg1 [F(3, 28) = 2.09, *p* > 0.05; Figure 9A] or PrL [F(3, 28) = 2.16, *p* > 0.05; Figure 9B].

**Figure 10:**
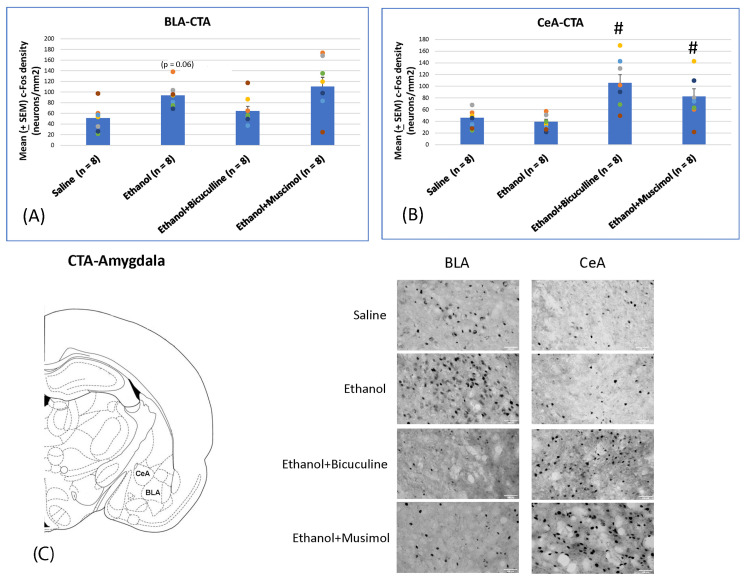
c-Fos density (neurons/mm^2^) in (**A**) BLA and (**B**) CeA in the GABA system experiment for the Saline, Ethanol, Ethanol + Bicuculline, and Ethanol + Muscimol groups (n = 8 per group) following ethanol-induced aversion in CTA. (**C**) Representative cartoon brain map illustrating the locations of BLA and CeA (**left panel**) and corresponding immunohistochemical staining images showing c-Fos expression (**right panel**). (#) indicates significant differences compared to the Ethanol group.

The BLA revealed c-Fos expression after ethanol-induced CTA learning among all groups [F(3, 28) = 5.61, *p* < 0.05]. The Ethanol group showed a numerically higher c-Fos expression than the Saline group (*p* = 0.06; Figure 10A). On the other hand, the CeA showed significant differences in c-Fos expression for all groups [F(3, 28) = 9.25, *p* < 0.05] by one-way ANOVA analysis; c-Fos expression was significantly increased in the Ethanol + Bicuculline and Ethanol + Muscimol groups compared to the Ethanol group (*p* < 0.05; Figure 10B) following post hoc Tukey tests.

**Figure 11:**
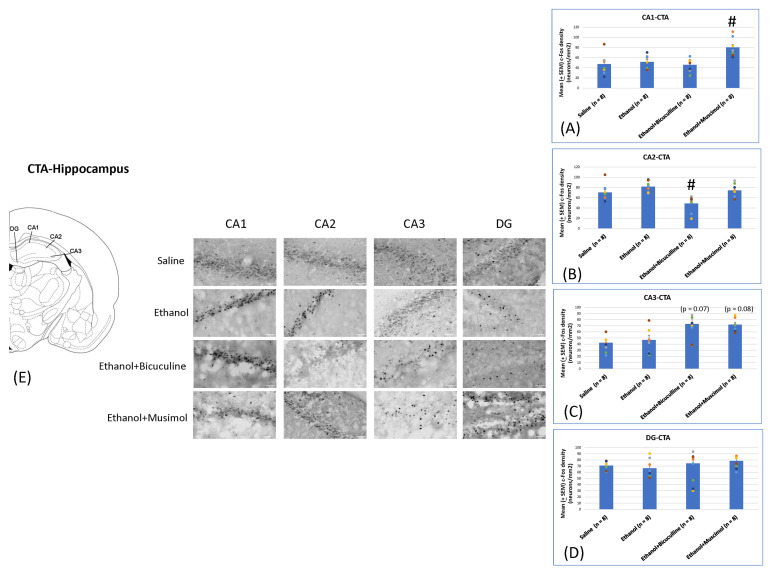
c-Fos density (neurons/mm^2^) in (**A**) CA1, (**B**) CA2, (**C**) CA3, and (**D**) DG of the hippocampus in the GABA system experiment for the Saline, Ethanol, Ethanol + Bicuculline, and Ethanol + Muscimol groups (n = 8 per group) following ethanol-induced aversion in CTA. (**E**) Representative cartoon brain map illustrating the locations of CA1, CA2, CA3, and DG (**left panel**) and corresponding immunohistochemical staining images showing c-Fos expression (**right panel**). (#) indicates significant differences compared to the Ethanol group.

To test c-Fos expression in the hippocampus after ethanol-induced aversion during CTA learning, the results revealed significant differences in the CA1 [F(3, 28) = 7.82, *p* < 0.05], CA2 [F(3, 28) = 8.37, *p* < 0.05], and CA3 [F(3, 28) = 5.23, *p* < 0.05]. There were significant increases in c-Fos expression in the CA1 for the Ethanol + Muscimol group compared to the Ethanol group (*p* < 0.05; Figure 11A). For the CA2, c-Fos expression was significantly decreased in the Ethanol + Bicuculline group compared to the Ethanol group (*p* < 0.05; Figure 11B). For the CA3, a numerical increase in c-Fos expression was observed in the Ethanol + Bicuculline (*p* = 0.07) and Ethanol + Muscimol (*p* = 0.08) groups compared to the Ethanol group (Figure 11C). However, no significant differences in c-Fos expression were observed in the DG (all, *p* = 0.73~0.99; Figure 11D).

#### 2.3.2. CPP Learning

**Figure 12:**
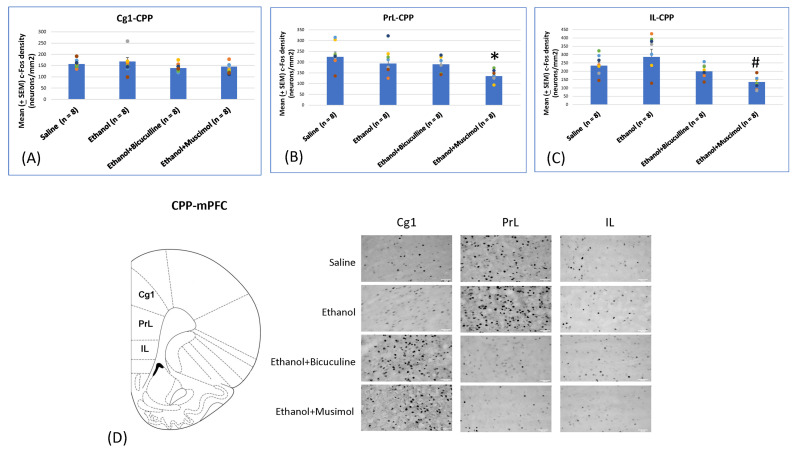
c-Fos density (neurons/mm^2^) in (**A**) Cg1, (**B**) PrL, and (**C**) IL of the mPFC in the GABA system experiment for the Saline, Ethanol, Ethanol + Bicuculline, and Ethanol + Muscimol groups (n = 8 per group) following ethanol-induced reward in CPP. (**D**) Representative cartoon brain map illustrating the locations of Cg1, PrL, and IL (**left panel**) and corresponding immunohistochemical staining images showing c-Fos expression (**right panel**). (*) indicates significant differences compared to the Saline group. (#) indicates significant differences compared to the Ethanol group.

The results showed that during CPP learning, the PrL had significant differences in c-Fos expression across all groups [F(3, 28) = 3.73, *p* < 0.05]. c-Fos expression was significantly decreased in the Ethanol + Muscimol group compared to the Saline group (*p* < 0.05); However, no significant differences occurred at the Ethanol + Bicuculline (*p* > 0.05) and Ethanol + Muscimol (*p* > 0.05) groups compared to the Ethanol (Figure 12B). Significant differences in c-Fos expression were observed in the IL for all groups [F(3, 28) = 5.36, *p* < 0.05]. c-Fos expression in the Ethanol + Muscimol group was significantly decreased compared to the Ethanol group (*p* < 0.05; Figure 12C). However, no significant differences in c-Fos expression were observed in the Cg1 [F(3, 28) = 1.19, *p* > 0.05; Figure 12A].

**Figure 13:**
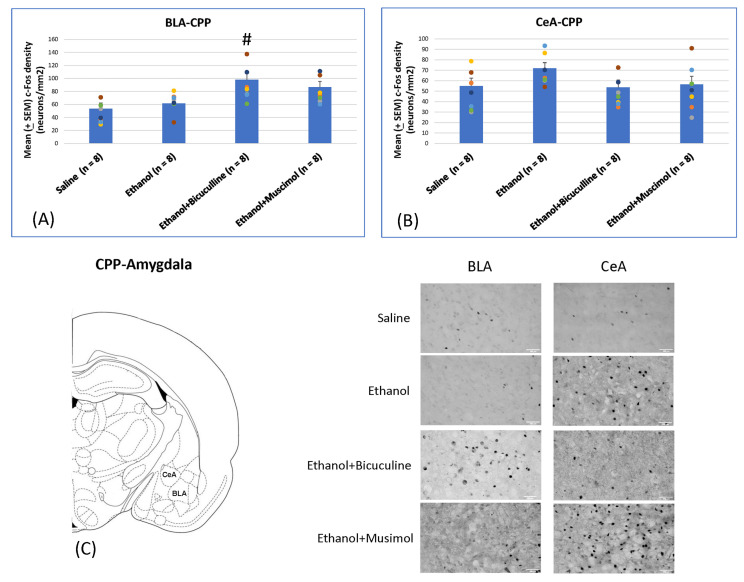
c-Fos density (neurons/mm^2^) in (**A**) BLA and (**B**) CeA in the GABA system experiment for the Saline, Ethanol, Ethanol + Bicuculline, and Ethanol + Muscimol groups (n = 8 per group). (**C**) Representative cartoon brain map illustrating the locations of BLA and CeA (**left panel**) and corresponding immunohistochemical staining images showing c-Fos expression (**right panel**). (#) indicates significant differences compared to the Ethanol group.

To assess c-Fos expression in the BLA and CeA, the results indicated significant differences in c-Fos expression in the BLA for all groups [F(3, 28) = 5.35, *p* < 0.05]. The BLA showed a numerical increase for c-Fos expression for the Ethanol + Muscimol (*p* = 0.06) group and a significant increase for the Ethanol + Bicuculline group (*p* < 0.05) compared to the Ethanol group (Figure 13A). However, there were no significant differences in c-Fos expression in the CeA [F(3, 28) = 1.47, *p* > 0.05; Figure 13B].

**Figure 14:**
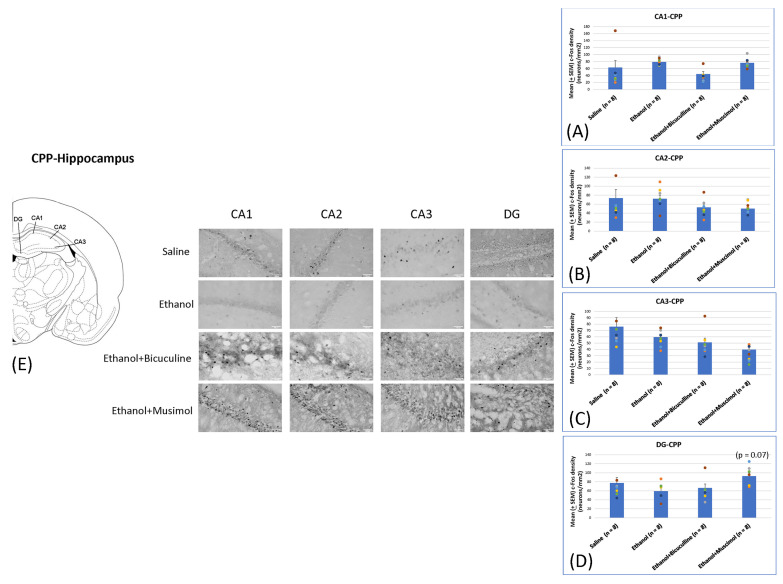
c-Fos density (neurons/mm^2^) in (**A**) CA1, (**B**) CA2, (**C**) CA3, and (**D**) DG of the hippocampus in the GABA system experiment for the Saline, Ethanol, Ethanol + Bicuculline, and Ethanol + Muscimol groups (n = 8 per group) following ethanol-induced reward in CPP. (**E**) Representative cartoon brain map illustrating the locations of CA1, CA2, CA3, and DG (**left panel**) and corresponding immunohistochemical staining images showing c-Fos expression (**right panel**).

To assess c-Fos expression in the subareas of the hippocampus during the CPP task, the results showed a numerical increase in c-Fos expression in the DG, although these did not reach statistical significance [F(3, 28) = 2.54, *p* > 0.05]. The Ethanol + Muscimol group showed a numerically higher c-Fos expression than the Ethanol group (*p* = 0.07; Figure 14D). However, no significant differences were observed in the CA1 [F(3, 28) = 2.06, *p* > 0.05; Figure 14A], CA2 [F(3, 28) = 1.21, *p* > 0.05; Figure 14B], or CA3 [F(3, 28) = 2.73, *p* > 0.05; Figure 14C].

### 2.4. Heatmap Analysis of c-Fos Expression in Various Brain Areas for CTA and CPP

**Figure 15:**
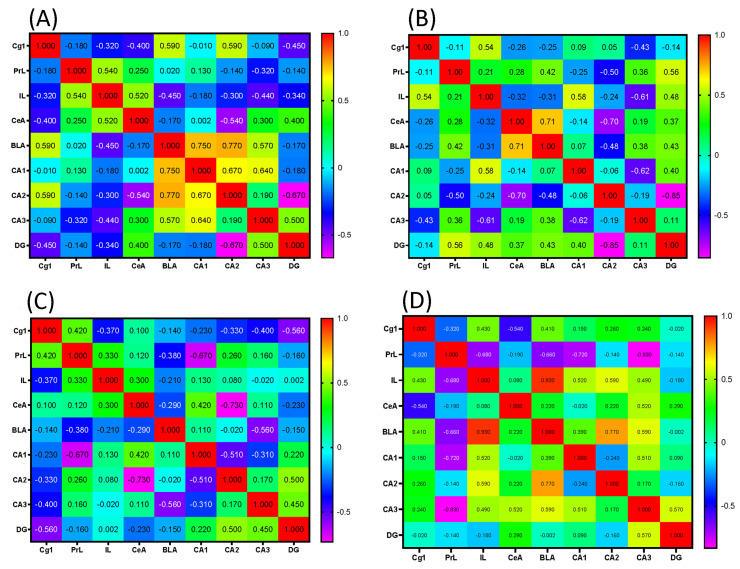
Heatmap displays Pearson’s correlation coefficients of c-Fos density (neurons/mm^2^) after ethanol-induced aversion in the conditioned taste aversion task in the GABA system experiment among brain regions, including the mPFC (Cg1, PrL, IL), amygdala (BLA, CeA), and hippocampus (CA1, CA2, CA3, DG) for the (**A**) Saline, (**B**) Ethanol, (**C**) Ethanol + Bicuculline, and (**D**) Ethanol + Muscimol groups (n = 8 per group). The matrix is presented to visualize the direction and magnitude of inter-regional associations; no correlations survived statistical significance testing. Long-wavelength colors indicate higher positive correlation, while short-wavelength colors indicate lower or negative correlation.

**Figure 16:**
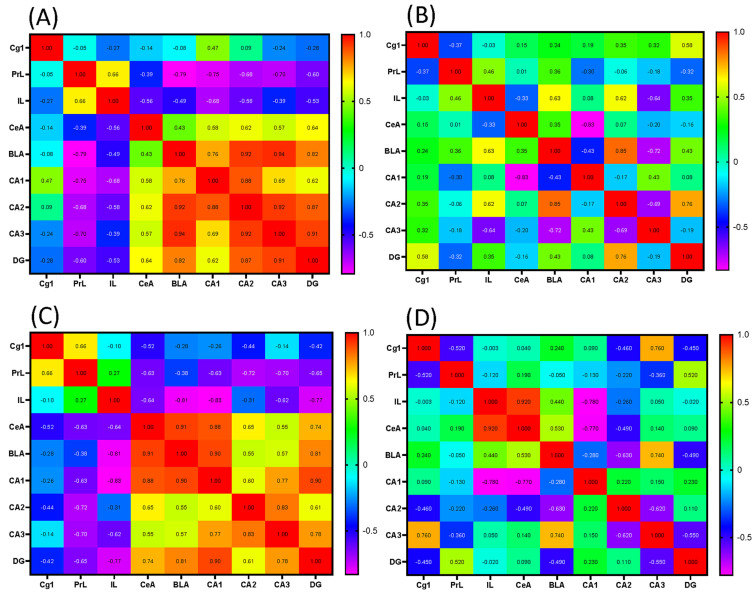
Heatmap displays Pearson’s correlation coefficients of c-Fos density (neurons/mm^2^) after ethanol-induced reward in the conditioned place preference task in the GABA system experiment among brain regions, including the mPFC (Cg1, PrL, IL), amygdala (BLA, CeA), and hippocampus (CA1, CA2, CA3, DG) for the (**A**) Saline, (**B**) Ethanol, (**C**) Ethanol + Bicuculline, and (**D**) Ethanol + Muscimol groups (n = 8 per group). The matrix is presented to visualize the direction and magnitude of inter-regional associations; no correlations survived statistical significance testing. Long-wavelength colors indicate higher positive correlation, while short-wavelength colors indicate lower or negative correlation.

These analyses are intended to visualize pharmacologically biased patterns of regional co-activation rather than infer network directionality or causality. The matrix of Figure 15 and Figure 16 is presented to visualize the direction and magnitude of inter-regional associations; no correlations survived statistical significance testing.

In Experiment 2, the CPP score (%) in the paired side was significantly decreased compared to the unpaired side in the Ethanol + Bicuculline and Ethanol + Muscimol groups. This prompted the question of whether the decreases in CPP score (%) for the Ethanol + Bicuculline and the Ethanol + Muscimol groups were reflected in distinct neural patterns. Thus, we analyzed and discussed c-Fos expression in the mPFC’s Cg1, PrL, and IL; the amygdala’s BLA and CeA; the hippocampus’ CA1, CA2, CA3, and DG using heatmaps to visualize differences in neural activity.

During the CTA task, the Ethanol group exhibited higher correlations between the mPFC’s PrL and the amygdala’s BLA (r = 0.42), the mPFC’s PrL and hippocampus’ DG (r = 0.56), and the mPFC’s IL and the hippocampus’ DG (r = 0.48). The amygdala’s BLA was more strongly correlated with the DG (r = 0.43). Overall, c-Fos expression in the Ethanol group demonstrated many more activity patterns among the subareas of the mPFC, amygdala, and hippocampus compared to the Saline group (Figure 15A,B).

Conversely, the Ethanol + Bicuculline group exhibited fewer activity patterns. For example, the subareas of the mPFC, such as the Cg1, PrL, and IL, showed negative or weaker correlations with the subareas of the amygdala, such as the CeA and BLA (i.e., all correlations were lower than r = 0.3). Furthermore, the mPFC’s Cg1, PrL, and IL were also negatively or more weakly correlated with the hippocampus (i.e., r < 0.03). There were negative or weaker correlations among the mPFC, amygdala, and hippocampus following injections of ethanol and bicuculline, the GABA(A) receptor antagonist. Thus, bicuculline could disrupt the activity patterns among the mPFC, amygdala, and hippocampus alongside a single ethanol injection (Figure 15C).

The Ethanol + Muscimol group exhibited higher c-Fos expression than the Ethanol group. For example, the mPFC’s Cg1 had higher positive correlations with the amygdala’s BLA (r = 0.41), and the mPFC’s IL was more strongly positively correlated with the BLA (r = 0.93). The mPFC’s IL was more strongly correlated with the hippocampus’ CA1 (r = 0.52), CA2 (r = 0.59), and CA3 (r = 0.49). The amygdala’s CeA showed higher correlations with the hippocampus’ CA3 (r = 0.52), and the amygdala’s BLA showed higher correlations with the hippocampus’s CA2 (r = 0.77) and CA3 (r = 0.59). Thus, positive correlations were found among the subareas of the mPFC, amygdala, and hippocampus (Figure 15D).

In summary, the Ethanol group displayed CTA learning associated with positive activity patterns among the subareas of the mPFC, amygdala, and hippocampus. Injections of GABA(A) receptor antagonist, bicuculline, disrupted the positive activity patterns among the mPFC, amygdala, and hippocampus. In contrast, the GABA(A) receptor agonist, muscimol, enhanced the positive activity patterns among the subareas of the mPFC, amygdala, and hippocampus. Note that the *p* values for the Saline, Ethanol, Ethanol + Bicuculline, and Ethanol + Muscimol groups after the CTA test are shown in Table S1 (see Table S1 in Supplementary Tables).

c-Fos expression in the mPFC (e.g., the Cg1, PrL, and IL), amygdala (e.g., BLA and CeA), and hippocampus (e.g., CA1, CA2, CA3, and DG) was analyzed, and the correlations were visualized using heatmaps following the CPP test. The results showed that in the Ethanol group, correlations between the subareas of the mPFC (e.g., Cg1, PrL, and IL) and the hippocampus’ CA1, CA2, CA3, and DG were stronger compared to those in the Saline group (Figure 16A,B). In particular, the Cg1 exhibited a greater positive correlation with the DG (r = 0.58), and the IL showed higher positive correlations with the CA2 (r = 0.62). In addition, the mPFC’s IL exhibited higher correlations with the amygdala’s BLA (r = 0.63). The amygdala’s BLA was highly positively correlated with the hippocampus’ CA2 (r = 0.85) and DG (r = 0.43; Figure 16B). Therefore, ethanol injections could enhance the activity patterns among the subareas of the mPFC, amygdala, and hippocampus.

Activity patterns between the subareas of the mPFC and the subareas of the amygdala and hippocampus were decreased in the Ethanol + Bicuculline group. However, the Ethanol + Bicuculline group showed higher positive correlations between the amygdala’s BLA and CeA and the hippocampus’ subareas (e.g., CA1, CA2, CA3, and DG). The correlation values were very high, ranging from 0.91 to 0.55 (Figure 16C). Thus, injections of ethanol and the GABA(A) receptor antagonist, bicuculline, enhanced the activity patterns between the amygdala and hippocampus but not the mPFC.

In the Ethanol + Muscimol group, the activity patterns among the mPFC, amygdala, and hippocampus were decreased compared to the Ethanol group (Figure 16B,D). For example, the correlation between the mPFC’s Cg1 and areas of the hippocampus decreased: CA1 (from r = 0.19 to r = 0.09), CA2 (from r = 0.35 to r = −0.46), and DG (from r = 0.58 to r = −0.45), with the exception of CA3 (from r = 0.32 to r = 0.76). This pattern also occurred in almost all hippocampal subareas with the PrL and IL (Figure 16B,D). Activity patterns between the mPFC’s Cg1, PrL, and IL and the amygdala’s BLA and CeA decreased. In the Ethanol + Muscimol group, the amygdala’s BLA and CeA showed consistent correlations with the hippocampal subareas compared to the Ethanol group (Figure 16D).

In conclusion, injections of ethanol and the GABA(A) receptor agonist, muscimol, exhibited decreased activity patterns among the subareas of the mPFC, amygdala, and hippocampus compared to the Ethanol group following the CPP tests. Note that the *p* values for the Saline, Ethanol, Ethanol + Bicuculline, and Ethanol + Muscimol groups after the CPP test are shown in Table S2 (see Table S2 in Supplementary Tables).

## 3. Discussion

The present study showed that a low dose of ethanol was administered and concurrently induced aversive learning in conditioned taste aversion (CTA) and rewarding learning in conditioned place preference (CPP), supporting the paradoxical effect hypothesis of abused drugs. Importantly, these aversive and rewarding effects were dissociable at both behavioral and neural levels through pharmacological manipulation of dopamine D2 and GABA(A) receptor systems. By combining CTA and CPP paradigms with c-Fos immunohistochemistry, the present findings extend prior neuropharmacological work by identifying receptor-specific and context-dependent engagement of corticolimbic circuits underlying ethanol-induced aversion and reward.

The ethanol dose used in the present study (0.20 g/kg) was intentionally selected to model the acute intoxication phase rather than withdrawal or chronic exposure. This dosage has been shown to reliably produce learning-related effects while minimizing nonspecific sedative or motor-suppressive confounds [1]. The observation that ethanol produced opposing behavioral outcomes across CTA and CPP paradigms argues against a generalized suppression of behavior and instead supports a learning-dependent modulation of motivational valence.

Although APO is not receptor-selective at high doses, extensive pharmacological evidence indicates that doses above 0.5 mg/kg predominantly activate postsynaptic dopamine receptors rather than presynaptic autoreceptors [23]. The present study employed a 20 mg/kg dose of APO to ensure robust dopaminergic activation at the circuit level, consistent with previous behavioral and neuropharmacological studies using similar dosage ranges [23,24,25].

Importantly, the behavioral effects observed following APO administration cannot be readily attributed to nonspecific motor impairment or sedation. APO enhanced ethanol-induced aversion in the CTA paradigm while simultaneously suppressing ethanol-induced reward and inducing conditioned place aversion in the CPP paradigm. This bidirectional modulation across distinct learning contexts indicates that dopaminergic activation selectively biases motivational processing rather than globally impairing performance. Thus, the present findings support a functional role of dopamine receptor activation—rather than receptor subtype selectivity per se—in regulating ethanol’s paradoxical aversive and rewarding effects during acute intoxication.

Although the CTA and CPP designs reduce the likelihood that the observed effects are solely attributable to nonspecific motor impairment or sedation, this possibility cannot be completely excluded. Therefore, an issue emerged of whether a 20 mg/kg high dose of APO induced hyperactivity and stereotyped behaviors should be scrutinized in further research.

In our study, although c-Fos does not directly reflect receptor-level signaling, it provides a useful index of pharmacologically induced circuit engagement following receptor manipulation. During the behavioral tests, ethanol injections induced paradoxical effects, simultaneously eliciting aversion in the CTA task and reward in the CPP task. These findings are consistent with those of previous studies [5,26] and further support the paradoxical effect hypothesis for abused drugs, including alcohol [1]. Importantly, the observed effects were dissociable across CTA and CPP paradigms, arguing against a nonspecific motor or sedative explanation.

At the dose used in the present study, APO predominantly activates postsynaptic dopamine receptors, consistent with previous pharmacological characterizations, rather than presynaptic autoreceptors. Our results demonstrate that the dopamine D2 agonist APO enhanced ethanol-induced aversive effects during the CTA test. Conversely, when co-administered with ethanol, APO diminished its rewarding effects, subsequently inducing a CPA effect. Despite several prior investigations, the precise mechanisms by which D1 and D2 receptors mediate ethanol’s rewarding and aversive properties remain debated. For example, the intraperitoneal injection of D1 receptor antagonist SCH23390 attenuated ethanol-induced CPP in one study; however, in another, the intraperitoneal injection of D2 receptor antagonist raclopride did not affect CPP induced by ethanol [13]. While chronic stressors such as tail pinch increase NAc dopamine release, suggesting the involvement of the mesolimbic dopamine system in responses to negative stimuli, acute ethanol administration has been shown to reduce NAc dopamine release [27], implicating this system in ethanol-associated aversion. Furthermore, a review paper reported that differential subpopulations of ventral tegmental area (VTA) dopamine neurons played different roles in the control of reward, aversion, and stress [28]. Therefore, the mesolimbic dopamine system is associated with both the aversion and the reward induced by ethanol. However, how to further dissociate the VTA-NAc pathway function in aversion CTA and reward CPP remains to be scrutinized in the next studies.

During the CTA test, co-administration of the GABA(A) receptor antagonist bicuculline with ethanol increased saccharin solution intake, while co-administration of the GABA(A) receptor agonist muscimol decreased it. This indicates that GABA(A) receptors modulate ethanol-induced aversion. Bicuculline or muscimol, administered with ethanol, both decreased CPP score (%), indicating that the GABA(A) receptor antagonist bicuculline reduced ethanol’s rewarding effect. The GABA(A) receptor agonist muscimol appeared to induce a CPA effect, potentially by overriding ethanol’s rewarding properties. Our findings related to the brain’s GABAergic system partially align with previously reported evidence [14], further supporting the notion that GABA(A) receptors interact with ethanol to mediate both rewarding and aversive effects [14,29]. It is important to note that different rodent species exhibit distinct ethanol effects [14]. For instance, data from mice have shown that a GABA(A) receptor antagonist enhanced ethanol-induced CPP and CTA, but rat data show a decrease in CTA [14]. Furthermore, the interplay between the dopamine and GABA systems plays a crucial role in AUD intoxication and binge [11].

Collectively, these behavioral findings suggest that both the dopaminergic and GABAergic systems play a critical role in modulating ethanol’s dual aversive and rewarding effects.

### 3.1. The Paradoxical Effect Hypothesis and Koob’s Three-Stage Cycle Hypothesis

Ethanol consumption can elicit both rewarding and aversive effects, which contribute to behaviors such as craving, vomiting, and avoidance, thereby influencing an individual’s susceptibility to alcohol consumption and the development of AUD [28,30]. Previous studies suggest that AUD results from the dysfunction of three-stage cycle phases: binge/intoxication, preoccupation/anticipation, and withdrawal/negative affect phases [7,31]. These three stages feed into one another, intensifying each other, finally leading to pathological AUD. This is termed Koob’s three-stage cycle hypothesis [7,32]. Koob’s hypothesis posits that reward acts as positive reinforcement during the preoccupation/anticipation or binge/intoxication phases, while individuals experience negative reinforcement during the withdrawal/negative affect phase [33,34]. The negative reinforcement is the outcome of the withdrawal phase [33]. However, this concept of negative reinforcement, primarily associated with withdrawal, is not directly parallel to the concept of aversion in the paradoxical effect hypothesis. The latter suggests that aversion and reward occur simultaneously during the acute intoxication phase, not necessarily during withdrawal [3]. Additionally, the concept of aversion is somewhat similar to the punishment property, based on the rules of behaviorism [35]. Therefore, the three-stage cycle hypothesis focuses on the positive reinforcement and negative reinforcement of preoccupation/anticipation or binge/intoxication and withdrawal/negative affect phases, respectively. In contrast, the paradoxical effect hypothesis emphasizes the rewarding and aversive properties of abused drugs during the acute intoxication phase but not the withdrawal phase.

Therefore, the paradoxical effect hypothesis, focusing on acute intoxication, presents a distinct perspective from Koob’s three-stage cycle hypothesis, which emphasizes positive reinforcement during intake and negative reinforcement primarily during withdrawal in the context of AUD. From a neuropharmacological perspective, the present findings emphasize receptor-specific modulation of aversive and rewarding states during intoxication, rather than withdrawal-related negative reinforcement.

### 3.2. Modulation of the Dopamine and GABA Systems in the mPFC, Amygdala, and Hippocampus by Ethanol-Induced Reward and Aversion

The present study’s c-Fos data showed that c-Fos density in only the BLA of the amygdala (but not the mPFC and the hippocampus) was significantly increased following ethanol injections in CTA but not CPP tests. The present data did not fully support the previous evidence. That is probably because we used the low dose of ethanol injections in the CTA and CPP test. For example, converging lines of evidence demonstrate that ethanol administration disrupts the neural activity of the mPFC and impairs amygdala inhibition [15,36]. Acute ethanol intoxication inhibited the neural activity of the mPFC, leading to cognitive impairments [37].

On the other hand, the present results showed that dopamine and GABA systems modulated ethanol-induced reward CPP and aversion in CTA (Supplementary Material, Tables S3 and S4). The results supported the previous evidence. For example, functional MRI data demonstrate a positive association between prefrontal cortex impairment and cognitive dysfunctions in patients with AUD, suggesting that they may struggle to effectively regulate cravings and negative emotional responses [16]. Moreover, mPFC impairment and CeA hyperactivity induce excessive drinking [15]. AUD patients exhibited functional connectivity between the amygdala and the prefrontal cortex for alcohol cues compared to neutral cues [38], indicating that the mPFC and amygdala are both involved in AUD and negative emotional stimuli. Regarding alterations in the hippocampus following ethanol administration, acute and chronic ethanol consumption impaired spatially related learning and memory [39] and neural plasticity [19]. Additionally, acute ethanol consumption was shown to alter the hippocampus and related structures [20]. Collectively, these findings highlight that AUD severity is associated with dysfunctions in the mPFC, amygdala, and hippocampus. Our c-Fos data extend previous findings by elucidating the differential involvement of the dopamine and GABAergic systems within specific subregions of the mPFC, amygdala, and hippocampus in mediating ethanol-induced aversion during the CTA task.

The present study’s results revealed that injections of dopamine agonist APO alongside ethanol significantly increased c-Fos expression in the amygdala’s BLA and CeA and the hippocampus’ CA1, indicating that APO augmented neural activity in the amygdala and specific hippocampal subregions, thereby modulating ethanol-induced aversive effects in CTA. In contrast, GABA(A) receptors were also involved in ethanol-induced aversion in CTA. To illustrate this, the GABA(A) receptor antagonist bicuculline, injected with ethanol, decreased c-Fos expression in the IL of the mPFC and the CA2 of the hippocampus. However, there were increases in c-Fos expression in the CeA of the amygdala compared to the Ethanol group. GABA(A) receptor agonist, muscimol, injected with ethanol, showed that c-Fos expression was robustly increased in the IL of the mPFC, amygdala’s CeA, and the hippocampal CA1 compared to the Ethanol group. The present data on the dopamine agonist APO indicate that it enhances neural activity via the amygdala’s BLA and CeA and the hippocampal CA1 following CTA tests. The GABA(A) receptor antagonist bicuculline showed hypoactivity in the mPFC’s IL and the hippocampus’ CA2; moreover, bicuculline was associated with hyperactivity in the CeA of the amygdala and the CA3 of the hippocampus under ethanol-induced aversive effects during CTA. The GABA(A) receptor agonist, muscimol, enhanced neural activity in the CeA of the amygdala and the CA1 of the hippocampus following ethanol’s aversive effects in CTA (Supplementary Material, Table S3).

On the other hand, after ethanol-induced CPP, APO showed a numerical increase in c-Fos expression in the PrL of the mPFC and the DG of the hippocampus, but decreased c-Fos expression in the CeA of the amygdala, indicating that the dopamine system modulated ethanol-induced reward in CPP by upregulating neural activity in the PrL of the mPFC and the DG of the hippocampus but downregulating neural activity in the CeA of the amygdala. Moreover, GABA(A) receptor antagonist, bicuculline, decreased c-Fos expression in the IL of the mPFC, while increasing c-Fos expression in the BLA of the amygdala following ethanol’s rewarding effects in CPP. Injections of GABA(A) receptor agonist, muscimol, probably increased c-Fos expression in the DG of the hippocampus following ethanol-induced reward in CPP. These results indicate that GABA(A) receptors upregulate neural activity in the DG of the hippocampus (Supplementary Material, Table S4).

In summary, the subareas of the mPFC, amygdala, and hippocampus made distinct contributions to the ethanol-induced aversion in CTA and reward in CPP during the ethanol intoxication phase.

### 3.3. Heatmap Analysis Insights

These heatmap analyses do not reflect functional connectivity but rather pharmacologically reorganized patterns of coordinated neural activity following receptor manipulation. In the present study, GABA(A) receptor antagonist, bicuculline, and GABA(A) agonist, muscimol, when co-administered with ethanol, both induced a CPA effect at the behavioral level, as indicated by negative CPP scores (%).

However, these groups exhibited different neural and organizational patterns in c-Fos expression tests. For example, the heatmap analysis showed that the Ethanol + Bicuculline group had decreased activity patterns between the mPFC’s Cg1, PrL, and IL and the hippocampus’ CA1, CA2, CA3, and between the DG and the amygdala’s CeA and BLA. However, the amygdala’s CeA and BLA activity patterns with the hippocampus’ CA1, CA2, CA3, and DG increased. In contrast, the Ethanol + Muscimol groups showed decreased activity patterns among the subareas of the mPFC, amygdala, and hippocampus; however, activity patterns in certain brain areas were increased (e.g., Cg1 and CA3; PrL and DG; BLA and CA3). This suggests that the GABA(A) receptor antagonist bicuculline disrupted the activity patterns between the mPFC and the hippocampus and increased the activity patterns between the amygdala and the hippocampus.

Conversely, the GABA(A) receptor agonist muscimol broadly attenuated activity patterns among the mPFC, amygdala, and hippocampus, although with selective increases in certain pairs (e.g., Cg1 and CA3; PrL and DG; BLA and CA3).

### 3.4. Experimental Limitations and Emerging Issues

The present study raised several issues, as follows. For example, this study aims to examine whether D2 agonist APO and GABA(A) agonist, muscimol, and GABA(A) receptor antagonist, bicuculline, could affect ethanol-induced aversion in CTA and reward in CPP. Thus, the study did not test whether GABA(A) receptor antagonist, bicuculline, and GABA(A) agonist, muscimol, themselves could induce CPP and CTA conditioning. Moreover, the present study did not examine whether the D2 agonist, APO, itself could induce CPP and CTA. This issue should be examined in further studies. Second, the previous studies demonstrated that the VTA was involved in reinforcement [22,40] and aversion [41], and the lateral habenula governed the aversive information and projected to the medial portion of the VTA [41]. Therefore, whether the lateral habenula and VTA were involved in behavioral mechanisms that the D2 receptor and GABA(A) receptors modulated ethanol-induced CTA and CPP remains unclear and needs to be scrutinized. Third, the study did not elucidate the sexual differences in the involvement of D2 and GABA(A) receptors in ethanol-induced CTA and CPP. Based on the previous evidence, the ethanol addiction might be induced by the sexual differences in males and females [42,43,44]. Therefore, the female rats should be considered for testing in further study. Fourth, the present data revealed that dopamine agonist APO could facilitate ethanol-induced aversion in CTA. The result was inconsistent with the previous evidence [12,45,46]. In the previous studies, D2 antagonist (haloperidol) or D1 antagonist (SCH 23390) could impair ethanol-induced aversion in CTA; however, the reduced effect was based on the dosages and conditioned flavor [12]. Moreover, the dopamine agonist, cocaine, attenuated ethanol-induced aversion in CTA. Nevertheless, an opposite study showed that a D3 antagonist, U99194A, did not affect the acquisition of ethanol-induced CTA [46]. Why did the present data conflict with the previous evidence? The reasons result from the different species (rat vs. mouse) or the dosage of ethanol. The emerging issue warrants further investigation. Finally, the used dose (i.e., 0.2 g/kg) of ethanol was referred to in our previous study [1]. In the previous CTA studies, the doses of ethanol were used from 1.2 to 4 g/kg [12,45,46]. The present dose of ethanol was significantly lower than that in the previous studies. However, the present study showed the ethanol-induced aversion in the CTA task, similar to our previous one [1]. In particular, the present study required conditioning with the saccharin solution for at least 3–4 sessions, and the ethanol-induced aversion in CTA was achieved. During the early 1–2 sessions, the ethanol could not induce aversion in CTA. Thus, the low dose of ethanol (0.2 g/kg) could also induce aversion in CTA.

Recently, some studies suggested that the basal ganglia were involved in action selection and reward preferences [47,48]. In the study, the basal ganglia were not tested in the immunohistochemical staining with c-Fos protein after ethanol-induced aversion in CTA and reward CPP tests. Thus, this issue should be examined in future research.

### 3.5. Conclusions

Previous research has explored novel pharmacological treatments for AUD and alcoholism. For instance, a review paper suggested that the stimulation of opioid kappa-receptors blocks the acute reinforcement and rewarding effects and induces aversive and dysphoric-like effects. The reinforcement blockade possibly results from the punishment/aversive-like effects, indicating that the opioid kappa-receptors modulate AUD symptoms [49]. Another approach focuses on the balance of ethanol-induced aversion and reward in the VTA [50]. This review paper suggested that acute and chronic ethanol exposure in the VTA induced neuroadaptation, leading to reduced aversion, thereby increasing the reward for imbalance due to AUD and alcoholism [50]. Thus, recovering the balance between reward and aversion should be considered in the development of novel therapeutic approaches. The third viewpoint is using oxytocin therapy to treat AUD and alcoholism [51]. Central or peripheral oxytocin administration could decrease ethanol intake, according to human and animal evidence. Alternatively, oxytocin receptors have been demonstrated to be expressed by dopamine neurons from the VTA to brain areas that mediate the aversive effect; however, oxytocin receptors are also expressed by non-dopamine neurons in the VTA, including GABA and glutamate neurons, which modulate ethanol-induced positive reinforcement (reward) or aversion [51]. Thus, oxytocin can be developed as a novel pharmacotherapy to treat AUD.

In the present study, we provide the fourth viewpoint to inform future studies on alcohol use disorder and valence-related mechanisms during the intoxication phase. In light of the present data, the dopamine and GABA systems can be considered in the development of novel pharmacotherapy. For example, dopamine agonist APO enhanced neural activity in the amygdala’s BLA and CeA and the hippocampus’ CA1 for ethanol-induced aversion in CTA. Thus, dopamine antagonists in the above brain areas are likely to attenuate ethanol-induced aversion in CTA. In contrast, our data showed that the GABA system is involved in the alteration of neural activity in the IL, the CeA of the amygdala, and the CA1 and CA2 of the hippocampus. Manipulating neural activity in the IL, BLA, CeA, CA1, and CA2 could inform pharmacological strategies for AUD and alcoholism, especially for ethanol-induced aversion in CTA.

On the other hand, our data showed that alterations of neural activity in the PrL, CeA, and DG can attenuate ethanol-induced reward in CPP following the administration of the dopamine agonist APO, underlying the dopamine system. Moreover, the GABA system is involved in the changes in the IL, BLA, and DG in the ethanol-induced reward in CPP. Therefore, changes in neural activity in the PrL, IL, BLA, CeA, and DG might represent mechanistic implications for pharmacotherapy development based on the present findings. Rather than serving as a comprehensive network analysis, these findings highlight how dopamine and GABAergic receptor manipulations bias corticolimbic circuit engagement toward aversive or rewarding learning during intoxication.

In summary, our findings highlight the mPFC, amygdala, and hippocampus, along with their associated dopamine and GABAergic systems, as crucial targets for the development of potential pharmacological targets aimed at rebalancing ethanol’s aversive and rewarding properties to treat AUD and alcoholism.

## 4. Materials and Methods

### 4.1. Animals

Eighty male Sprague–Dawley (SD) rats, weighing 250–300 g (approximately 8 weeks) at the beginning of the study, were purchased from BioLASCO Biotechnology Co.,Yilan County, Taiwan. Rats were housed in pairs in plastic cages under a controlled light–dark cycle (lights on at 6:00 AM, lights off at 6:00 PM) with stable room temperature. Food and water were offered ad libitum, except where specific water deprivation procedures were implemented. The experiment adhered to American Psychological Association guidelines and received approval from the Fo Guang University Institutional Animal Care and Use Committee. The number of animals used was limited, and every effort was made to minimize animal suffering during the experiment.

### 4.2. Apparatuses

#### 4.2.1. Lickometer

The lickometer consisted of a wire-mesh cage, a white panel, and a 25 mL burette graduated in 0.1 mL increments. The burette was connected to the white panel and mounted above the wire-mesh cage. The intake volume of fluid collected for each trial served as the raw data.

#### 4.2.2. Conditioned Place Preference

The CPP box was a T-shaped apparatus comprising two approximately square wooden chambers (45 × 43 × 43 cm height) and a small intermediary shuttle compartment (38 × 18 × 18 cm height). The three compartments were separated by wood partitions. The two square preference chambers were accessible via the shuttle compartment, and each featured a clear Plexiglas observation wall on one side. One of the preference compartments was white with a wire-grid floor, and the other was painted with white/black stripes with a wood-chip bedding floor. The raw data for the CPP task included the time spent in the paired and unpaired compartments for each group.

### 4.3. Experimental Procedure

**Figure 17:**
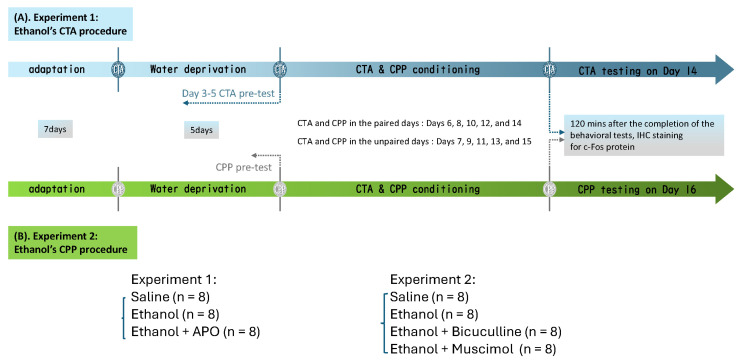
Experimental procedure for ethanol-induced conditioned taste aversion (CTA) and conditioned place preference (CPP). This figure outlines the experimental design for (**A**) Experiment 1, testing the dopamine system, and (**B**) Experiment 2, investigating the GABA system. All rats underwent a 7-day adaptation period, followed by 5 days of water deprivation, 10 days of CTA and CPP conditioning, and 1 day of CTA/CPP testing. Two hours following behavioral testing, c-Fos protein immunohistochemistry was performed in specific brain regions, including the medial prefrontal cortex (mPFC) subregions (Cg1, PrL, and IL), the amygdala (BLA and CeA), and hippocampal subfields (CA1, CA2, CA3, and DG). During water deprivation, all rats received a CTA pre-test (Days 3–5) to adapt to the lickometer device or a CPP pre-test (Day 5) to adapt to the CPP box. Experiment 1 included the Saline, Ethanol, and Ethanol + APO groups (n = 8 per group). Experiment 2 consisted of the Saline, Ethanol, Ethanol + Bicuculline, and Ethanol + Muscimol groups (n = 8 per group). Note: Cg1, cingulate cortex 1; PrL, prelimbic cortex; IL, infralimbic cortex; BLA, basolateral amygdala; CeA, central amygdala; DG, dentate gyrus; APO, apomorphine.

This study comprised two experiments: Experiment 1 investigated the dopamine system, and Experiment 2 investigated the GABA system in relation to alcohol-induced aversion in CTA and reward in CPP. At the beginning of Experiments 1–2, all rats experienced a 7-day habituation period in their home cages, during which they had ad libitum access to food and water. Subsequently, the CTA baseline measurement was conducted over three days (Days 3–5), followed by one day of the CPP baseline measurement (Day 5). With the exception of the adaptation phase, all rats were subjected to water deprivation for 23.5 h per day, followed by 30 min of access to water in the evening in their home cage. During the conditioning phase (even-numbered days), rats received 0.1% saccharin solution for 15 min, followed by an intraperitoneal injection of 0.20 g/kg ethanol or normal saline. Subsequently, rats were placed into one of the components of the CPP box for 30 min. On the odd-numbered days of the conditioning phase, all rats remained in their home cages without receiving a drug injection, and then received an intraperitoneal injection of normal saline for 30 min. Then the rats were placed into another component of the CPP box. The conditioning phase lasted 10 days in total (Days 6–14). Ethanol administration was paired with both CTA and CPP learning for five trials (i.e., on Days 6, 8, 10, 12, and 14). Then, the rats proceeded to the testing phase for the CTA test in Experiment 1 (Figure 17A) and the CPP test in Experiment 2 (Figure 17B). CTA testing was given on Day 14 and CPP testing on Day 16. During the testing phase, rats first underwent the CTA procedure for 15 min without drug administration, followed by the CPP procedure for 15 min. CTA and CPP learning effects were assessed during this period. Two hours (120 min) after completion of the behavioral tests, the animals were sacrificed. Immunohistochemical staining was performed to label c-Fos protein expression in specific brain regions, including the Cg1, PrL, IL, CeA, BLA, CA1, CA2, CA3, and DG (Figure 17).

#### 4.3.1. Experiment 1: Dopamine System

Experiment 1 was designed to determine whether the dopamine system was involved in ethanol-induced aversion (CTA) and reward (CPP). All rats were randomly assigned to one of three groups: Saline (n = 8), Ethanol (n = 8), or Ethanol + APO (20 mg/kg; n = 8). APO was intraperitoneally injected with ethanol on the even days of the conditioning phase for the Ethanol + APO group. Based on the existing literature, APO acts as a DA antagonist at low doses (<0.1 mg/kg), activating dopamine autoreceptors. At high doses (>0.5 mg/kg), it binds with postsynaptic receptors, acting as a DA agonist [23]. Therefore, the dose of 20 mg/kg APO used in this study functioned as a dopamine receptor agonist.

#### 4.3.2. Experiment 2: GABA System

Experiment 2 was designed to investigate whether the GABA system was involved in the ethanol-induced aversion (CTA) and reward (CPP). All rats were randomly assigned to one of four groups: Saline (n = 8), Ethanol (n = 8), Ethanol + Bicuculline (n = 8), or Ethanol + Muscimol (n = 8). Bicuculline and muscimol were intraperitoneally injected with ethanol on the even-numbered days of the conditioning phase for the Ethanol + Bicuculline and Ethanol + Muscimol groups. Bicuculline served as a GABA(A) receptor antagonist, and muscimol as a GABA(A) receptor agonist [52]. Note that the behavioral and histochemical staining data of the Saline and Ethanol groups in Experiment 2 shared the same data as in Experiment 1.

### 4.4. Immunohistochemical Staining

Rats were sacrificed by sodium pentobarbital overdose. When completely unresponsive, rats were perfused with 100 mL 0.9% NaCl followed by 400 mL of 4% paraformaldehyde in 0.1 M sodium phosphate-buffered saline (PBS). The brain tissue was dissected, blocked, post-fixed for 3 days, and transferred to 30% sucrose for cryoprotection for 2 days until the specimens sank to the bottom of the solution. Fifty-micron coronal sections were cut on a freezing sliding microtome. All sections were performed by c-Fos immunoreactivity staining. Free-floating brain sections were washed once for 10 min in 0.1 M PBS, permeabilized in 3% H_2_O_2_ for 1 h, washed three times in PBS for 10 min, and then soaked in normal goat serum for 1 h. After washing with PBS once for 10 min, the sections were incubated overnight with the first antibody [rabbit anti-c-Fos antibody at a dilution of 1:1000 (Millipore, ABE457)]. This antibody was raised against a peptide sequence to enable cross-reaction with other proteins for detecting c-Fos immunoreactivity. The section was washed once with PBS for 10 min and incubated in a second antibody [biotinylated goat anti-rabbit antibody at a dilution of 1:500 (Vector Lab BA-1000)] for 2 h. After a 10 min PBS wash, the bound secondary antibody was amplified using the Vector Elite ABC kit (Vector Lab ABC Kit, PK-6100).

The positive expression of the c-Fos protein within neurons was quantified using ImageJ 1.53 software for each brain site. Counting was performed visually at 20 × magnification for each section of the brain tissue. Every third section of each brain was selected for counting. Every count of the brain section was averaged for each group. Moreover, c-Fos density positive neurons were analyzed by the following formula: c-Fos numbers/the section area (0.90 mm × 0.72 mm ≈ 0.648 mm^2^).

### 4.5. Drugs

Absolute ethanol and sodium chloride were purchased from Sigma-Aldrich (St. Louis, MO, USA). The ethanol dose used in this study was 0.20 g/kg, prepared by diluting 100% (*w*/*v*) absolute ethanol in normal saline. The normal saline solution was prepared by dissolving sodium chloride (NaCl) in distilled water. Saccharin powder was diluted in distilled water to create a 0.1% saccharin solution. The 0.20 g/kg ethanol dose was selected based on our previous publication (He et al., 2017) [1]. The 20 mg/kg APO dose was selected based on previous studies [23,24,25], and the 0.5 mg/kg Bicuculline and 1 mg/kg muscimol doses were also selected based on a previous study [52]. All injections were administered intraperitoneally at a volume of 1 mL/kg.

### 4.6. Statistics

We performed a mixed two-way ANOVA on the intake volume of the saccharin solution to investigate the CTA effect. The saccharin solution was then analyzed using a one-way ANOVA for each session. When appropriate, Tukey post hoc tests were performed. To investigate the CPP effect, mean (±SEM) time spent in the unpaired and paired compartments was analyzed using a dependent *t*-test among all groups in the Baseline and Test phases. To account for individual differences, the mean (±SEM) time spent on the CPP task was transformed into CPP scores (%) using the following formula [53].

CPP score (%) = 100 * (Time spent in the paired side–Time spent in the unpaired side)/(Time spent in the unpaired side + Time spent in the paired side).

The CPP score (%) was analyzed by one-way ANOVA. To test mean (±SEM) c-Fos expression numbers, a one-way ANOVA was performed for all groups. When appropriate, post hoc Tukey tests were performed. (*) indicates significant differences at *p* < 0.05, and (n.s.) indicates that differences were nonsignificant. Tukey post hoc tests were applied based on a priori hypotheses and limited group numbers, a statistical approach commonly adopted in hypothesis-driven neuropharmacological studies to maintain sensitivity for detecting biologically meaningful effects [21].

## Data Availability

The data presented in this study are available at https://www.dropbox.com/scl/fo/ouw7mrsvg6w7beab64xaw/AGtLrQ8PO_VlvK4HtuTIu5E?rlkey=0wslg6v5k9sz1t8t7bjpcxin3&st=rrvjp372&dl=0 (accessed on 24 May 2026).

## Supplementary Materials

The following supporting information can be downloaded at: https://www.mdpi.com/article/10.3390/ijms27114987/s1.
