# Supplementary material for: Dopamine D2 and GABA(A) Receptors Differentially Regulate Ethanol-Induced Aversion and Reward Through Corticolimbic Circuits
Source: Int J Mol Sci. 2026 May 30;27(11):4987. doi: 10.3390/ijms27114987 (PMC13256999; doi:10.3390/ijms27114987)
Supplement: Supplementary file 1 [file ijms-27-04987-s001.zip › ijms-4290197-supplementary.pdf]

Supplementary Tables

Table S1. The p value in each cell for heatmap analysis of c-Fos expression correlations after ethanol-induced aversion (CTA) in the GABA system experiment. Pearson correlation analysis of c-Fos expression (number of positive cells) between subregions of the mPFC (Cg1, PrL, IL), amygdala (BLA, CeA), and hippocampus (CA1, CA2, CA3, DG) for the (A) Saline, (B) Ethanol, (C) Ethanol+Bicuculline, and (D) Ethanol+Muscimol groups (n = 8 per group). Note: mPFC, medial prefrontal cortex; Cg1, cingulate cortex 1; PrL, prelimbic cortex; IL, infralimbic cortex; BLA, basolateral amygdala; CeA, central amygdala; DG, dentate gyrus; CTA, conditioned taste aversion.

(A). Saline group

	Cg1	PrL	IL	CeA	BLA	CA1	CA2	CA3	DG
Cg1	0.00	--	--	--	--	--	--	--	--
PrL	0.68	0.00	--	--	--	--	--	--	--
IL	0.45	0.17	0.00	--	--	--	--	--	--
CeA	0.33	0.55	0.18	0.00	--	--	--	--	--
BLA	0.13	0.96	0.27	0.69	0.00	--	--	--	--
CA1	0.98	0.75	0.67	1.00	0.03	0.00	--	--	--
CA2	0.12	0.75	0.47	0.17	0.02	0.07	0.00	--	--
CA3	0.84	0.44	0.27	0.48	0.14	0.09	0.65	0.00	--
DG	0.27	0.75	0.41	0.32	0.68	0.67	0.07	0.21	0.00

(B). Ethanol group

	Cg1	PrL	IL	CeA	BLA	CA1	CA2	CA3	DG
Cg1	0.00	--	--	--	--	--	--	--	--
PrL	0.79	0.00	--	--	--	--	--	--	--
IL	0.17	0.62	0.00	--	--	--	--	--	--
CeA	0.54	0.50	0.44	0.00	--	--	--	--	--
BLA	0.56	0.30	0.45	0.05	0.00	--	--	--	--
CA1	0.83	0.55	0.13	0.74	0.87	0.00	--	--	--
CA2	0.90	0.20	0.57	0.06	0.23	0.88	0.00	--	--
CA3	0.29	0.39	0.11	0.66	0.35	0.11	0.65	0.00	--
DG	0.75	0.15	0.23	0.36	0.29	0.32	0.01	0.80	0.00

(C). Ethanol+Bicuculline group

	Cg1	PrL	IL	CeA	BLA	CA1	CA2	CA3	DG
Cg1	0.00	--	--	--	--	--	--	--	--
PrL	0.31	0.00	--	--	--	--	--	--	--
IL	0.37	0.43	0.00	--	--	--	--	--	--
CeA	0.82	0.78	0.47	0.00	--	--	--	--	--
BLA	0.74	0.35	0.61	0.49	0.00	--	--	--	--
CA1	0.59	0.07	0.77	0.30	0.79	0.00	--	--	--
CA2	0.42	0.53	0.84	0.04	0.96	0.20	0.00	--	--
CA3	0.33	0.70	0.97	0.79	0.15	0.45	0.69	0.00	--
DG	0.15	0.70	1.00	0.59	0.73	0.61	0.21	0.26	0.00

(D). Ethanol+Muscimol group

	Cg1	PrL	IL	CeA	BLA	CA1	CA2	CA3	DG
Cg1	0.00	--	--	--	--	--	--	--	--
PrL	0.44	0.00	--	--	--	--	--	--	--
IL	0.29	0.07	0.00	--	--	--	--	--	--
CeA	0.17	0.65	0.84	0.00	--	--	--	--	--
BLA	0.32	0.08	0.00	0.60	0.00	--	--	--	--
CA1	0.72	0.04	0.19	0.96	0.34	0.00	--	--	--
CA2	0.54	0.74	0.13	0.61	0.02	0.56	0.00	--	--
CA3	0.58	0.01	0.22	0.19	0.12	0.20	0.69	0.00	--
DG	0.97	0.74	0.68	0.46	1.00	0.83	0.71	0.14	0.00

Table S2. The p value in each cell for heatmap analysis of c-Fos expression correlations after ethanol-induced reward (CPP) in the GABA system experiment. Pearson correlation analysis of c-Fos expression (number of positive cells) between subregions of the mPFC (Cg1, PrL, IL), amygdala (BLA, CeA), and hippocampus (CA1, CA2, CA3, DG) for the (A) Saline, (B) Ethanol, (C) Ethanol+Bicuculline, and (D) Ethanol+Muscimol groups (n = 8 per group). Note: mPFC, medial prefrontal cortex; Cg1, cingulate cortex 1; PrL, prelimbic cortex; IL, infralimbic cortex; BLA, basolateral amygdala; CeA, central amygdala; DG, dentate gyrus; CPP, conditioned place preference.

(A). Saline group

	Cg1	PrL	IL	CeA	BLA	CA1	CA2	CA3	DG
Cg1	0.00	--	--	--	--	--	--	--	--
PrL	0.91	0.00	--	--	--	--	--	--	--
IL	0.52	0.07	0.00	--	--	--	--	--	--
CeA	0.75	0.34	0.15	0.00	--	--	--	--	--
BLA	0.85	0.02	0.22	0.29	0.00	--	--	--	--
CA1	0.24	0.03	0.06	0.13	0.03	0.00	--	--	--
CA2	0.83	0.06	0.14	0.10	0.00	0.00	0.00	--	--
CA3	0.56	0.06	0.34	0.14	0.00	0.06	0.00	0.00	--
DG	0.50	0.12	0.18	0.09	0.01	0.10	0.01	0.00	0.00

(B). Ethanol group

	Cg1	PrL	IL	CeA	BLA	CA1	CA2	CA3	DG
Cg1	0.00	--	--	--	--	--	--	--	--
PrL	0.36	0.00	--	--	--	--	--	--	--
IL	0.94	0.25	0.00	--	--	--	--	--	--
CeA	0.72	0.98	0.42	0.00	--	--	--	--	--
BLA	0.57	0.39	0.09	0.39	0.00	--	--	--	--
CA1	0.66	0.47	0.86	0.01	0.29	0.00	--	--	--
CA2	0.39	0.89	0.10	0.87	0.01	0.69	0.00	--	--
CA3	0.43	0.67	0.09	0.63	0.05	0.29	0.06	0.00	--
DG	0.13	0.45	0.40	0.70	0.29	0.85	0.03	0.65	0.00

(C). Ethanol+Bicuculline group

	Cg1	PrL	IL	CeA	BLA	CA1	CA2	CA3	DG
Cg1	0.00	--	--	--	--	--	--	--	--
PrL	0.07	0.00	--	--	--	--	--	--	--
IL	0.81	0.52	0.00	--	--	--	--	--	--
CeA	0.19	0.09	0.09	0.00	--	--	--	--	--
BLA	0.50	0.35	0.01	0.00	0.00	--	--	--	--
CA1	0.54	0.10	0.01	0.00	0.00	0.00	--	--	--
CA2	0.27	0.04	0.45	0.08	0.16	0.11	0.00	--	--
CA3	0.75	0.05	0.10	0.16	0.14	0.03	0.01	0.00	--
DG	0.31	0.08	0.03	0.04	0.02	0.00	0.11	0.02	0.00

(D). Ethanol+Muscimol group

	Cg1	PrL	IL	CeA	BLA	CA1	CA2	CA3	DG
Cg1	0.00	--	--	--	--	--	--	--	--
PrL	0.19	0.00	--	--	--	--	--	--	--
IL	0.99	0.78	0.00	--	--	--	--	--	--
CeA	0.93	0.66	0.00	0.00	--	--	--	--	--
BLA	0.57	0.91	0.27	0.18	0.00	--	--	--	--
CA1	0.84	0.75	0.02	0.03	0.50	0.00	--	--	--
CA2	0.25	0.60	0.53	0.21	0.09	0.60	0.00	--	--
CA3	0.03	0.38	0.91	0.74	0.04	0.72	0.10	0.00	--
DG	0.26	0.19	0.97	0.83	0.22	0.59	0.79	0.16	0.00

Table S3. c-Fos density in the mPFG's Cg1, PrL, and IL; amygdala's BLA and CeA; and hippocampus' CA1, CA2, CA3, and DG following the CTA test.

	mPFC			Amygdala		Hippocampus			
	Cg1	PrL	IL	BLA	CeA	CA1	CA2	CA3	DG
**Saline (n = 8)**	--	--	--	--	--	--	--	--	--
**Ethanol (n = 8)**	--	--	--	*(↑)	--	--	--	--	--
**Ethanol+APO (n = 8)**	--	--	--	#(↑)	#(↑)	#(↑)	--	--	--
**Ethanol+Bicuculline (n = 8)**	--	--	#(↓)	--	#(↑)	--	#(↓)	--	--
**Ethonal+Muscimol (n = 8)**	--	--	--	--	#(↑)	#(↑)	--	--	--

Note: mPFC: medial prefrontal cortex; Cg1: cingulate cortex; PrL: prelimbic cortex; IL: infralimbic cortex; BLA: basolateral amygdala; CeA: central amygdala; DG: dentate gyrus; CTA: conditioned taste aversion; (*): significant differences compared to the Saline group; (#): significant differences compared to the Ethanol group; (↑): increases; (↓): decreases.

Table S4. c-Fos density in the mPFG's Cg1, PrL, and IL; amygdala's BLA and CeA; and hippocampus' CA1, CA2, CA3, and DG following the CPP test.

	mPFC			Amygdala		Hippocampus			
	Cg1	PrL	IL	BLA	CeA	CA1	CA2	CA3	DG
**Saline (n = 8)**	--	--	--	--	--	--	--	--	--
**Ethanol (n = 8)**	--	--	**--**	**--**	--	--	--	--	--
**Ethanol+APO (n = 8)**	--	#(↑)	--	--	#(↓)	--	--	--	#(↑)
**Ethanol+Bicuculline (n = 8)**	--	--	#(↓)	#(↑)	--	--	--	--	--
**Ethonal+Muscimol (n = 8)**	--	--	--	#(↑)	--	--	--	--	#(↑)

Note: mPFC: medial prefrontal cortex; Cg1: cingulate cortex; PrL: prelimbic cortex; IL: infralimbic cortex; BLA: basolateral amygdala; CeA: central amygdala; DG: dentate gyrus; CPP: conditioned place preference; (*): significant differences compared to the Saline group; (#): significant differences compared to the Ethanol group; (↑): increases; (↓): decreases.
